# Robotic surgery in the interior of Brazil: Is it possible?

**DOI:** 10.1590/0100-6991e-20253838-en

**Published:** 2025-11-24

**Authors:** JORGE ROBERTO MERCANTE-CARLOTTO, PETRA MISTURA ARCOVERDE CAVALCANTI, ANA LUÍSA DOS SANTOS CARREGOSA, EDUARDA LEITZKE, LARA FABIAN DE MOURA, MILENA DE ALMEIDA DA MOTTA, NICOLE MOMBELLI MATTEI, RODRIGO GUERRA-CASARIN, TAMI ZANG CRESTANI

**Affiliations:** 1 - Universidade de Passo Fundo, Medicina - Passo Fundo - RS - Brasil; 2 - Hospital de Clínicas de Passo Fundo - Passo Fundo - RS - Brasil

**Keywords:** General Surgery, Robotic Surgery, Operative Complications, Urology, Cirurgia Geral, Cirurgia Robótica, Complicações Operatórias, Urologia

## Abstract

**Introduction:**

Robotic surgery has been used in the treatment of various surgical diseases due to its precision and satisfactory outcomes. This study aims to describe the profile of patients undergoing robotic surgery at the Hospital de Clínicas de Passo Fundo and analyze the variables related to the procedure and its outcomes.

**Methods:**

A total of 215 medical records of patients who underwent robotic surgery at the Regional Robotic Surgery Center of the Hospital de Clínicas de Passo Fundo were reviewed, from the start of the program in 2023 until March 2024. Sex, age, comorbidities, and perioperative and postoperative data were evaluated.

**Results:**

The sample had a predominance of males (73.5%). The average age was 61 years. Systemic arterial hypertension was the most prevalent comorbidity (43.7%). Regarding operative time, the first six months had a higher median total time (300 minutes) compared to the last six months (245 minutes) with p≤0.001.

**Conclusion:**

The implementation of a robotic surgery center in the interior of Brazil proved to be feasible, with favorable outcomes, progressive reductions in surgical times, and regional benefits by expanding access to cutting-edge technologies.

## RESUMO

**Introdução:**

A cirurgia robótica tem sido utilizada no tratamento de diversas doenças cirúrgicas devido à sua precisão e resultados satisfatórios. Este estudo objetiva descrever o perfil dos pacientes submetidos à cirurgia robótica no Hospital de Clínicas de Passo Fundo e analisar as variáveis relacionadas ao procedimento e seus desfechos.

**Métodos:**

Foram revisados 215 prontuários de pacientes submetidos à cirurgia robótica no Centro Regional de Cirurgia Robótica do Hospital de Clínicas de Passo Fundo desde o início do programa em 2023 até março de 2024. Foram avaliados sexo, idade, comorbidades e dados operatórios e pós-operatórios.

**Resultados:**

A amostra teve predominância do sexo masculino (73.5%). A média de idade foi de 61 anos. A hipertensão arterial sistêmica foi a comorbidade mais prevalente (43,7%). A urologia concentrou 66,5% dos procedimentos, com destaque para prostatectomia radical. A taxa global de complicações foi de 17,7%. Quanto ao tempo operatório, os primeiros seis meses obtiveram uma mediana maior de tempo total (300 minutos) em comparação com os últimos seis meses (245 minutos) com p≤0,001.

**Conclusão:**

A implantação de um centro de cirurgia robótica no interior do Brasil mostrou-se viável, com desfechos favoráveis, redução progressiva dos tempos cirúrgicos e benefícios regionais ao ampliar o acesso a tecnologias de ponta.

## INTRODUCTION

The term robot, first proposed in the early twentieth century, although innovative, brings the already old idea of autonomous machines. In this sense, the first robot capable of imitating human movements was proposed by Leonardo da Vinci in the fifteenth century^1^. In the surgical area, the main robots are dependent on the surgeon’s actions, called master-slave systems^2^. In this context, the Da Vinci surgical system, launched in the 1990s, shows a great evolution in the current health environment^3^. 

Robotic surgery consists of a procedure performed by videosurgery, with a minimally invasive technique, in which all maneuvers are conducted by a qualified surgeon through a remote control over the robotic arms. The advantages of the technique include high-quality images, better depth perception, and improved amplitude and precision of movement of instruments4. However, this technology is still little available in Brazil, especially in cities with less than 300,000 inhabitants.

From this perspective, it is essential to outline the epidemiological profile of patients undergoing robotic surgery, as well as to evaluate the team, the procedure, and other factors associated with this technology in a medium-sized city in the interior of Brazil. These data can encourage and assist the implementation of this technology in other municipalities for the benefit of the population that lives outside large centers.

## METHODS

The present study was approved by the Ethics in Research Committee of the University of Passo Fundo under Opinion 6.559.049. The study is a retrospective review of the database of the Regional Center for Robotic Surgery of the Hospital de Clínicas de Passo Fundo (HCPF), state of Rio Grande do Sul, in Southern Brazil. 

From March 8, 2023 to March 8, 2024, 223 robotic surgeries were performed, and 215 medical records, both electronic and physical, were reviewed. Eight patients were excluded from the study due to incomplete information in their medical records. Inclusion criteria included patients who underwent robotic surgeries consecutively in the period analyzed, if they had complete electronic and physical medical records. Cases whose documentation was incomplete or lacked relevant information for the proposed analyses were excluded. The period of analysis chosen was the first year after the implementation of the robotic surgery program at the Hospital de Clínicas de Passo Fundo, with the objective of evaluating the results of this technology in a city in the interior of the state of Rio Grande do Sul.

The variables analyzed were grouped into three categories:

- Demographic and clinical variables: sex, age, comorbidities, and disease treated;
- Operative variables: number of arms and clamps used in the Da Vinci X robot; in addition to the following times related to the procedure;
- Draping time - from the beginning of the organization of the sterile field to the complete installation of the robotic equipment;
- Anesthetic induction time - from the beginning of anesthesia administration to patient stabilization;
- Patient positioning time - necessary to adjust the patient to the appropriate surgical position;
- Portal-placement time - referring to the introduction of robotic instrument portals;
- Console time - duration of the surgeon’s active intervention on the console;
- Total operative time - from the beginning to the end of surgery;
- Postoperative outcomes: complication rate (in the immediate and late postoperative period), need for ICU admission, and time until the release of the oral diet.

The collected data were organized in Excel software (Microsoft^®^) spreadsheets and later exported for statistical analysis in the Statistical Package for the Social Sciences (SPSS), version 24.0 (SPSS Inc, Chicago, IL, USA). The Kolmogorov-Smirnov normality test was applied to numerical variables to verify whether they followed a normal distribution, which determined the choice between parametric statistical tests, in case of normality, or non-parametric ones, if not.

Descriptive statistics were presented as median (P25-P75) for continuous variables and absolute frequency (%) for categorical ones. To compare non-normally distributed numerical variables, we used the non-parametric Mann-Whitney U test. For categorical variables, we applied the chi-square as the standard, though we preferred the Fisher’s exact test in small sample situations or when expected frequencies were less than five, due to its higher accuracy in such conditions. Values of p≤0.05 were considered statistically significant.

## RESULTS

The sample consisted of 215 patients, mostly male (73.5%), with a mean age of 61.5 years. Only 13% of patients came from the city of Passo Fundo and the rest came from other municipalities or states. Table 1 summarizes the age distribution of the sample. More than half of patients (58.6%) had comorbidities, the most prevalent being systemic arterial hypertension (43.7%), dyslipidemia (12.2%), and diabetes mellitus (10.7%), as detailed in Table 1. 

**Table 1:** Demographic and clinical characteristics of the cohort.

	No. of patients	Percentage (%)*
Sex		
Male	158	73.5
Female	57	26.5
Age group (Age**)		
17-30 years		2.8
31-40 years		4.2
41-50 years old		13.5
51-60 years	45	20.9
61-70 years old		33.5
71-80 years	47	21.9
81-87 years	7	3.3
Origin		
Passo Fundo	28	13
Another municipality in RS		64.7
Other state	48	22.3
Comorbidities		
No	89	41.4
Yes	126	58.6
Hypertension		43.7
Dyslipidemia		12.1
Diabetes Mellitus	23	10.7
Hypothyroidism	17	7.9
Cardiovascular Diseases	12	5.6
Psychiatric Illnesses		5.1
Metabolic Disorders	11	5.1
Genitourinary Diseases	9	4.2
Lung Diseases		2.3
Gastric Diseases		2.3
Neurological Diseases		1.9
Rheumatological Diseases	4	1.9
Otorhinolaryngological Diseases	3	1.4
Other	10	4.7

*Percentage in relation to the total number of patients, 215. **Age: mean = 61.15 years, standard deviation = 13.22 years, median = 63 years, Q25 = 53.5 years, Q75 = 70.5 years, and variance = 174.77.

Urology concentrated most procedures (66.5%), followed by general surgery/digestive system (27.4%), oncology (2.8%), thoracic surgery (1.9%), and gynecology (1.4%). Malignant neoplasm of the prostate was the main surgical indication (47%). The complete distribution of specialties is shown in Table 2. Complications took place in 38 surgeries (17.7%). Table 3 shows the number of patients with associated complications. Of the 215 patients, 44 (20.5%) required admission to the intensive care unit (ICU). Regarding the reintroduction of diet in the postoperative period, 104 patients (48.6%) received food on the day of surgery, of whom 80.8% had undergone urological surgery, 101 patients (47.2%) had diet resumed on the first postoperative day, while only nine (4.2%) did so from the second day onwards. These data are presented in Table 3.

**Table 2:** Surgical distribution by specialties and procedure.

	No. of patients	Percentage (%)*
Specialty		
Urology	143	66.5
General Surgery/Digestive System	59	27.4
Gynaecology	3	1.4
	No. of patients	Percentage (%)*
Thoracic Surgery	4	1.9
Oncological Surgery	6	2.8
Procedure		
Radical Prostatectomy	109	50.7
Urinary Tract Surgery	29	13.5
Lower Gastrointestinal Tract Surgery	20	9.3
Upper Gastrointestinal Tract Surgery	12	5.6
Fundoplication	11	5.1
Hepatectomy	9	4.2
Pancreatic Surgeries	5	2.3
Other Abdominal Surgeries	5	2.3
Female Genital Tract Surgeries	4	0
Thoracic Surgeries	4	1.9
Hernioplasty	4	1.9
Cystectomy	3	1.4

*Percentage in relation to the total number of patients, 215.

**Table 3:** Postoperative outcomes.

	No. of patients	Percentage (%)*
Surgical complications		
No complication	177	82.3
With complications***	38	17.7
1 associated complication	22	57.9**
2 associated complications	11	28.9**
3 associated complications	4	10.5**
6 associated complications	1	2.6**
Need for ICU		
Yes	44	20.5
No	171	79.5
ICU Discharge Day		
Day 0	12	27.3
Day 1	20	45.5
Day 2	6	13.6
Day 3	2	4.5
Day 4	1	2.3
Day 5	3	6.8
Ambulation Day		
Day 0	16	7.4
Day 1	160	74.4
Day 2	22	10.2
Day 3 or more	7	3.3
Lost Data	10	4.7
Postoperative Diet Reintroduction		
Day 0	104	48.4
	No. of patients	Percentage (%)*
Day 1	101	47
Day 2 or more	9	4.2
Lost Data	1	0.5

*Percentage in relation to the total number of patients, 215. **Percentage of patients with complications, 38 patients. Complications: Respiratory, gastrointestinal, gastrourinary tract, changes in vital signs, changes in blood pressure and others (thrombocytopenia, thrombosis, allergy, delirium and death).

Regarding the total operative time, 62.5% of surgeries lasted between 201 and 350 minutes. The console time of 68.7% of procedures was between 101 and 250 minutes. Almost 90% of operations had draping time less than or equal to 15 minutes. In the comparison between the first and second semesters, there was a statistically significant reduction in draping times (p≤0.001), instrumental organization (p<0.03), anesthetic induction (p≤0.001), console time (p<0.047), and total operative time (p<0.001). The complication rate also dropped in the period, from 23.1% in the first semester to 12.1% in the second (p<0.049). The values detailed by semester are presented in Table 4 and Table 5.

**Table 4:** Chronological comparison of surgical times..

Surgical times	1^st^ semester (minutes)	2^nd^ semester (minutes)
Draping	11 (3-30)	10 (4-20)
Organization	15 (6-70)	14 (5-40)
Anesthetic induction	20 (1-79)	15 (1-40)
Positioning	16 (2-124)	14 (2-52)
Placement of portals	12 (3-47)	13 (2-71)
Console	194 (8-725)	165 (1-370)
Total	300 (104-993)	245 (135-564)

*Representation: A (B-C), where A is the median, B is the minimum time and C is the maximum time. All times are expressed in minutes. 1st semester from 14/03/23 to 19/09/23 and 2nd semester from 19/09/23 to 08/03/24.

**Table 5:** Chronological comparison of the number of surgical complications.

Complications	1st semester (number of patients)	2nd semester (number of patients)
No complications	83 (76.9%)	94 (87.9%)
With complications	25 (23.1%)	13 (12.1%)

*Representation: D (E%), where D is the number of patients and E is the percentage in relation to the total number of surgeries in the analyzed period (1st semester = 108 and 2nd semester = 107).

## DISCUSSION

The results clearly demonstrate that robotic surgery is feasible in the interior of Brazil, including in centers outside capitals. In just over a year and a half, 215 procedures were performed with mostly favorable outcomes, confirming the possibility of implementation and the relevance of the technique for the population of a macro-region. It is noteworthy that most patients came from neighboring municipalities, evidencing the overcoming of distance barriers and the expansion of access to a technology previously restricted to large urban centers. This scenario highlights the positive impact of the decentralization of highly complex resources, which reduces inequalities in access to health and promotes a more equitable standard of care. 

The learning curve seen over a year since the beginning of robotic surgeries in the analyzed service was remarkable. This fact is based on the evident reduction in surgical times in the second semester of the study, both in specific times, such as draping and console, and in the average total procedure time. Another factor is the occurrence of complications, which were also less common in the group evaluated in the second half of the study. A study conducted at the Faculty of Medicine of the Pontifical Catholic University of Paraná showed similar results, with an average reduction of 73 minutes in total surgical time (approximately 25%), in addition to an optimization of one minute (33%) and 18 minutes (9%) in docking and console time, respectively8. In the present study, we also observed a significant decrease in the average number of clamps used, as well as a reduction in the average number of team members, suggesting improvement of technique and execution over time. International studies ranging from minimally invasive pancreatectomy to inguinal hernia repair have also shown a trend towards improved performance and optimization of robotic surgery after the learning curve period^11^. 

Our study demonstrated a profile like that of the national literature: male patients, mostly in the age group of 60 years, and hypertensive, with a predominance of urological procedures5. The international literature, although scarce in relation to the epidemiological characteristics, presents a profile in the age group of 46.9 years in the field of colorectal robotic surgery, inguinal hernia repair surgery, and bariatric surgery by Roux-en-y gastric bypass^7^ ^,^ ^8^. In view of the epidemiological data, an association with cardiovascular complications and higher surgical risk is inferred and assumed, which may directly influence outcomes, such as prolonged surgical time, need for intensive hemodynamic monitoring, or higher incidence of postoperative complications. 

The present study has limitations, such as its retrospective nature, that is, it is based entirely on the collection of existing data, with the outcome already determined at the time of collection and the possibility of lack of reliable data or even limited access to these data. However, the control of this limitation was done through the training of the data collection team and review of the complete medical records of each patient, both electronic and physical. Moreover, there is no control group, there being no two patients for whom the only different factor is the surgical approach, being impossible to isolate this factor as beneficial or deleterious with 100% accuracy. There is still the absence of follow-up, which makes it impossible to evaluate long-term complications.

The main highlight of this study is being the first in Brazil to report the initial experience with robotic surgery in a city in the interior. These data can be useful for the formation of new robotic surgery centers outside Brazilian capitals, especially in the countryside, providing broad access to high-quality healthcare.

## CONCLUSION

Robotic surgery in the interior of Brazil has proven to be feasible and safe, with a predominance of male patients, over 60 years of age, and with comorbidities. Radical prostatectomy was the most performed procedure, with a low need for ICU, early recovery, and a low complication rate. There was a significant reduction in surgical times in the last six months, reflecting the team’s learning curve. The installation of the center in Passo Fundo expanded the access of patients from other municipalities and states (87% of the sample), contributing to decentralizing high-complexity services and reducing inequalities in access to specialized health.
