# Erratum

**DOI:** 10.1590/0100-6991e-202638383erratum-en

**Published:** 2026-05-22

The article “Robotic surgery in the interior of Brazil: Is it possible?”, DOI number: 10.1590/0100-6991e-20253838-en, published in the Journal of the Brazilian College of Surgeons 52:e20253838.

Where it is: 

Authorship

Jorge Roberto Mercante-Carlotto TCBC-RS- 0000-0001-5769-6123

Afiliação(ões): [1] - Universidade de Passo Fundo, Medicina - Passo Fundo - RS - Brasil

[2] - Hospital de Clínicas de Passo Fundo - Passo Fundo - RS - Brasil

Petra Mistura-Arcoverde-Cavalcanti - 0009-0006-7095-5349

Afiliação(ões): [1] - Universidade de Passo Fundo, Medicina - Passo Fundo - RS - Brasil

Ana Luísa Dos-Santos-Carregosa - 0000-0002-9993-2822

Afiliação(ões): [1] - Universidade de Passo Fundo, Medicina - Passo Fundo - RS - Brasil

Eduarda Leitzke - 0000-0002-9341-8881

Afiliação(ões): [1] - Universidade de Passo Fundo, Medicina - Passo Fundo - RS - Brasil

Lara Fabian-de-Moura - 0000-0001-8887-8851

Afiliação(ões): [1] - Universidade de Passo Fundo, Medicina - Passo Fundo - RS - Brasil

Milena De-Almeida-da-Motta - 0000-0003-3193-3340

Afiliação(ões): [1] - Universidade de Passo Fundo, Medicina - Passo Fundo - RS - Brasil

Nicole Mombelli Mattei - 0000-0001-6531-2417

Afiliação(ões): [1] - Universidade de Passo Fundo, Medicina - Passo Fundo - RS - Brasil

Rodrigo Guerra-Casarin - 0000-0003-0721-4397

Afiliação(ões): [2] - Hospital de Clínicas de Passo Fundo - Passo Fundo - RS - Brasil

Tami Zang-Crestani - 0000-0003-2985-1485

Afiliação(ões): [1] - Universidade de Passo Fundo, Medicina - Passo Fundo - RS - Brasil

It should be: 

Authorship

Jorge Roberto Mercante-Carlotto TCBC-RS- 0000-0001-5769-6123

Afiliação(ões): [1] - Universidade de Passo Fundo, Medicina - Passo Fundo - RS - Brasil

[2] - Hospital de Clínicas de Passo Fundo - Passo Fundo - RS - Brasil

Petra Mistura-Arcoverde-Cavalcanti - 0009-0006-7095-5349

Afiliação(ões): [1] - Universidade de Passo Fundo, Medicina - Passo Fundo - RS - Brasil

Ana Luísa Dos-Santos-Carregosa - 0000-0002-9993-2822

Afiliação(ões): [1] - Universidade de Passo Fundo, Medicina - Passo Fundo - RS - Brasil

Eduarda Leitzke - 0000-0002-9341-8881

Afiliação(ões): [1] - Universidade de Passo Fundo, Medicina - Passo Fundo - RS - Brasil

Eduardo Giongo - 0000-0003-2509-8414

Afiliação(ões): [1] - Universidade de Passo Fundo, Medicina - Passo Fundo - RS - Brasil

Fábio Augusto Scharnberg - 0000-0002-6112-9284

Afiliação(ões): [1] - Universidade de Passo Fundo, Medicina - Passo Fundo - RS - Brasil

Henrique Lorini-Ferrarin - 0000-0002-4559-168X

Afiliação(ões): [1] - Universidade de Passo Fundo, Medicina - Passo Fundo - RS - Brasil

Julia Krefta-Zanin - 0000-0002-2707-8671

Afiliação(ões): [1] - Universidade de Passo Fundo, Medicina - Passo Fundo - RS - Brasil

Lara Fabian-de-Moura - 0000-0001-8887-8851

Afiliação(ões): [1] - Universidade de Passo Fundo, Medicina - Passo Fundo - RS - Brasil

Milena De-Almeida-da-Motta - 0000-0003-3193-3340

Afiliação(ões): [1] - Universidade de Passo Fundo, Medicina - Passo Fundo - RS - Brasil

Nicole Mombelli Mattei - 0000-0001-6531-2417

Afiliação(ões): [1] - Universidade de Passo Fundo, Medicina - Passo Fundo - RS - Brasil

Rodrigo Guerra-Casarin - 0000-0003-0721-4397

Afiliação(ões): [2] - Hospital de Clínicas de Passo Fundo - Passo Fundo - RS - Brasil

Tami Zang-Crestani - 0000-0003-2985-1485

Afiliação(ões): [1] - Universidade de Passo Fundo, Medicina - Passo Fundo - RS - Brasil

